# Thirty-Day Outcomes following Pediatric Bone and Soft Tissue Sarcoma Surgery: A NSQIP Pediatrics Analysis

**DOI:** 10.1155/2020/1283080

**Published:** 2020-02-14

**Authors:** Kathryn E. Gallaway, Junho Ahn, Alexandra K. Callan

**Affiliations:** Department of Orthopaedic Surgery, UT Southwestern Medical Center, Dallas, TX, USA

## Abstract

**Background:**

Pediatric bone and soft tissue sarcomas are rare; therefore, national registries are essential tools for orthopedic oncology research. Past studies provide excellent data on long-term prognosis and survival trends but fail to examine treatment-specific morbidity. The aim of this study is to use a national registry to describe patient demographics, comorbidities, and adverse events in the first thirty days following surgical management of pediatric bone and soft tissue sarcomas.

**Methods:**

A retrospective review of patients in the American College of Surgeons National Surgical Quality Improvement Program—Pediatrics database (NSQIP-P) was performed. The cohort was partitioned by tumor origin (bone versus soft tissue) and tumor location (axial versus appendicular).

**Results:**

One-hundred ninety-two patients were identified. Bone sarcomas were more common (71.9%) and predominately appendicular (62.3%), while soft tissue sarcomas were predominately axial (77.8%). The overall complication rate was 8.9%. The most frequent etiologies were wound dehiscence (3.6%) and infectious complications such as surgical site infections (2.6%), pneumonia (1.6%), urinary tract infections (1.6%), and *C. diff* colitis (1.0%). Twenty-four percent of patients experienced bleeding requiring transfusion. The unplanned readmission rate was 12.5% (3.6% related to principle procedure), and the unplanned reoperation rate was 4.7% (4.2% related to principle procedure). The mortality rate was 1.0%. Neoadjuvant chemotherapy was associated with higher rates of wound dehiscence and infectious complications. There were no differences in adverse events with respect to tumor origin or location.

**Conclusion:**

Approximately 1 in 11 pediatric patients will experience a complication in the first thirty days following surgery. However, perioperative mortality remains low. This study represents the first comprehensive review of pediatric bone and soft tissue sarcoma surgery in the NSQIP-P database. As the case volume of NSQIP-P continues to grow, NSQIP-P has the potential to become a powerful tool for pediatric orthopedic oncology research.

## 1. Introduction

Pediatric bone and soft tissue sarcomas are rare tumors associated with significant morbidity and long-term sequelae. The most common pediatric sarcomas are osteosarcoma, Ewing sarcoma, and rhabdomyosarcoma with a combined age-adjusted incidence of 2.7 cases per 100,000 children ages 0 to 19 in the United States [1]. Treatment of pediatric bone and soft tissue sarcoma is multidisciplinary, and treatment protocols vary by tumor location, histology, and stage at diagnosis [2]. Surgery is frequently used to obtain local control in patients with tumors amenable to resection, and there have been numerous advances in surgical techniques, imaging modalities, and adjuvant therapies leading to improvements in survival and functional outcomes [2–4]. Due to the rare incidence of these tumors, past research has frequently been limited to case series and small sample sizes [5–9].

International collaborations such as the European and American Osteosarcoma Study (EURAMOS-1) and national cancer registries such as the Surveillance, Epidemiology, and End Results program (SEER) have become essential research tools illustrating trends in epidemiology and survival in bone and soft tissue sarcomas [10–15]. These data are essential for determining the efficacy of treatment protocols and the incidence of long-term sequelae. However, a frequently cited limitation of SEER and other long-range studies is the lack of data on patient comorbidities and postoperative complications [13–20]. Understanding of treatment specific morbidity is an essential component of preoperative planning. Studies focusing on short-term outcomes can help identify patients who are most at risk of developing postoperative complications that may contribute to costly delays in adjuvant therapy.

The American College of Surgeons—National Surgical Quality Improvement Program (NSQIP)—is a national surgical registry prospectively collecting patient demographics, comorbidities, and complications in the first thirty days following surgery. Data collection methodologies and quality control measures for the NSQIP database have been previously described in detail [21]. The adult registry is well established with over six hundred participating sites across the United States and a current case volume of 6,638,405 procedures performed between 2005 and 2017. NSQIP has been used to characterize patient characteristics and outcomes after surgical management of primary and metastatic tumors of the spine [22, 23]. Our research team has also used NSQIP to study outcomes after surgical management of primary bone and soft tissue sarcomas and metastatic bone tumors of the extremities [24, 25].

In 2012, ACS opened enrollment for the NSQIP Pediatric (NSQIP-P) registry which has grown to include 109 hospitals across the United States with a current case volume of 483,098 procedures performed between 2012 and 2017. NSQIP-P has been used to describe adverse events following common pediatric orthopedic procedures in order to identify procedures and patient characteristics associated with an increased risk of postoperative complications [26]. Other studies have considered outcomes after specific orthopedic procedures [27–29], most frequently involving the spine [30–33], as well as outcomes in orthopedic patients with specific risk factors such as obesity, congenital heart disease, or cerebral palsy [34–36]. To our knowledge, NSQIP-P has not been used to characterize outcomes after pediatric orthopedic oncology procedures. The aim of this study is to describe patient demographics, comorbidities, surgical parameters, and adverse events in the first thirty days following surgical management of pediatric bone and soft tissue sarcomas.

## 2. Materials and Methods

After obtaining Institutional Review Board approval, a retrospective review of pediatric patients in NSQIP-P who underwent surgery for primary malignant bone or soft tissue sarcoma between 2012 and 2017 was performed. Patient demographics, comorbidities, surgical parameters, and adverse events were extracted. The cohort was partitioned by tumor origin (bone versus soft tissue) and tumor location (axial versus appendicular) to determine if there were any significant differences between these groups.

NSQIP-P data files for each year between 2012 and 2017 were combined into a single master file and queried using Apache Zeppelin 0.7.3 (Wakefield, MA). Patient data were extracted using International Classification of Diseases, Ninth Revision (ICD-9) and Tenth Revision (ICD-10) codes related to malignant neoplasm of bone, malignant neoplasm of connective and soft tissue, and malignant neoplasm of peripheral nerves. A complete list of ICD-9 and ICD-10 codes used to query the database is presented in [Supplementary-material supplementary-material-1]. This yielded an initial cohort of 621 patients.

Next, the cohort was refined using Current Procedure Terminology (CPT) codes to exclude patients undergoing procedures other than primary tumor management. Procedure codes used as inclusion criteria include bone or soft tissue biopsy, tumor ablation, excision of subcutaneous or subfascial tumor, radical resection of bone or soft tissue tumor, bone resection, soft tissue excision, osteoplasty, osteotomy, epiphyseal arrest, open reduction internal fixation, arthroplasty, arthrodesis, and external fixation and amputation. A comprehensive list of CPT codes used to query the database is presented in [Supplementary-material supplementary-material-1]. NSQIP-P provides one principle CPT code, up to ten other CPT codes (defined as an additional procedure performed under the same anesthesia by the same surgical team) and up to ten concurrent CPT codes (additional procedure performed by a different surgical team) for a total of twenty-one potential CPT codes per case. Cases with one or more of the identified procedures were included in the study. This narrowed the cohort to 197 patients.

**Figure 1:**
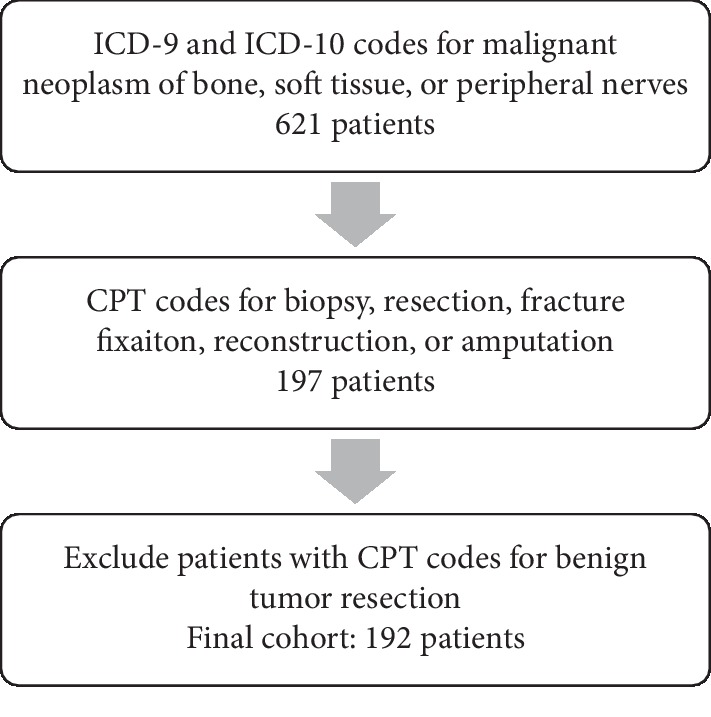
Cohort selection process.

Finally, patients with a CPT code for excision of benign tumor of bone or soft tissue were excluded from the study regardless of other diagnosis and procedure codes listed on the case. The list of CPT codes used as exclusion criteria is provided in [Supplementary-material supplementary-material-1]. From this selection process, 192 cases were identified and extracted for analysis (Figure 1).

The cohort was partitioned by tumor origin (bone versus soft tissue sarcoma) and tumor location (axial versus appendicular location) for subgroup analysis. Axial locations included the head, neck, and trunk, while appendicular locations included the upper extremity, shoulder girdle, lower extremity, and pelvis.

Primary outcome measures were the incidence of postoperative complications, perioperative transfusions, unplanned readmission, unplanned reoperation, and mortality. Rates of individual postoperative complications were calculated as secondary outcomes. Patient demographics, comorbidities and preoperative lab values, and surgical parameters were extracted and summarized. All patient factors, surgical parameters, and postoperative outcome measures were analyzed with respect to tumor origin and location.

Descriptive statistics were computed in Apache Zeppelin. Continuous variables were summarized with median and interquartile range (IQR) values. Categorical variables were reported as frequencies and percentages. Subgroups were compared using odds ratios and Fisher exact test for categorical variables or Pearson's *χ*^2^ test for variables with more than two categories. Continuous variables were compared using Mann–Whitney *U* test for nonparametric samples. An *α* value less than 0.05 was considered significant. Bivariate analysis was performed in GraphPad Prism 8.0.1 (San Diego, CA).

## 3. Results

**Figure 2:**
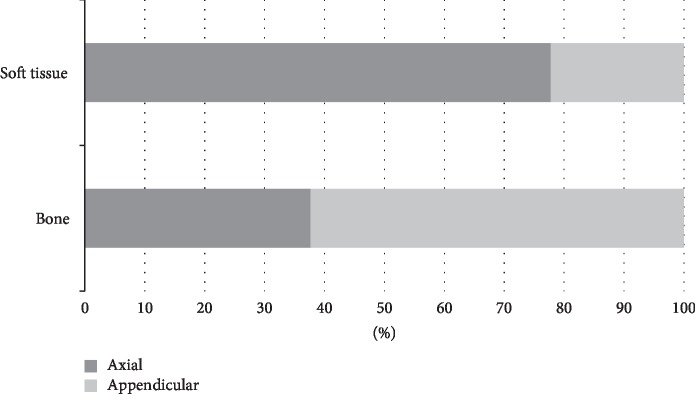
Relationship between tumor location and tumor origin.

One-hundred ninety-two patients were identified. Bone sarcomas were more common than soft tissue sarcomas (71.9% vs 28.1%), and tumors were evenly distributed between axial and appendicular locations. Bone sarcomas were predominately located in the appendicular skeleton (62.3%) while soft tissue sarcomas were predominately axial (77.8%) (Figure 2).

### 3.1. Demographics and Comorbidities

The median age of the study population was 11 years with a slight male predominance (Table 1). Bone sarcoma patients were older with a median age of 12 years compared with soft tissue sarcoma patients with a median age of 7.5 years (*p*=0.0006). Appendicular tumor patients were also older with a median age of 12 years versus 9 years in patients with axial tumors (*p* < 0.0001). African Americans made up a significantly larger proportion of soft tissue sarcoma patients (OR 4.626, 95% CI: 1.350–12.95). There were no other differences in patient demographics with respect to tumor location and origin (Table 2).

Comorbidities tracked in NSQIP-P include preoperative SIRS (1.6%), inotropic support (0.5%), nutritional support (4.2%), steroid use in the thirty days prior to surgery (12.0%), cardiac risk factors (4.2%), pulmonary disease (12.0%), gastrointestinal disease (3.6%), central nervous system disorder (13.5%), neuromuscular disorder (5.2%), hematologic disorder (13.5%), and history of prior childhood malignancy (8.9%) (Table 1). Patients with appendicular tumors were significantly more likely to have a history of prior childhood malignancy (OR 8.313, 95% CI: 2.146–37.250). NSQIP-P does not specify whether the current tumor is a recurrence of a prior malignancy or a second primary tumor. Axial tumors were significantly more likely to have a central nervous system disorder (OR 7.181, 95% CI: 2.419–19.850). There were no other statistically significant differences in comorbidities between subgroups (Table 2).

### 3.2. Preoperative Labs and Surgical Parameters

Median preoperative labs were all within the normal range. 34.9% of patients were classified as ASA 1 or 2 and 64.6% of patients were classified as ASA 3 or 4. The median duration of anesthesia was 303.5 minutes. The median length of hospital stay was 5 days. One appendicular bone sarcoma patient was given spinal anesthesia, but all other patients received general anesthesia. Eighty-three point three percent of surgeries were performed on an inpatient basis and 7.8% of cases were considered emergent or urgent (Table 3). The most frequent surgical modality was soft tissue or bony resection with no hardware or joint reconstruction (79.2%). Two appendicular bone tumors resulted in an amputation, with an overall amputation rate of 1.0%.

There were no differences in preoperative lab values or surgical parameters with respect to tumor origin, but there were significant differences between axial and appendicular tumors. Axial tumors had significantly higher median preoperative white blood cell counts (7.0 vs. 5.9, *p*=0.0044) and platelet counts (270.0 vs. 216.0, *p*=0.0010) compared with appendicular tumors. Axial procedures were also significantly more likely to be emergent or urgent (OR 16.98, 95% CI: 2.660–181.9). There were no other differences in surgical variables with respect to tumor location (Table 4).

### 3.3. Postoperative Complications

The most frequent complication was wound dehiscence. Superficial wound dehiscence occurred in 2.6% of patients and deep wound dehiscence in 1.0% of patients. Surgical site infections (SSIs) were the next most common complication, with superficial SSIs occurring in 1.6% of patients and deep SSIs in 1.0% of patients. Pneumonia, urinary tract infection (UTI), and seizure each occurred in 1.6% of patients. *C. diff* colitis occurred in 1.0% of patients. Nerve injury, graft/prosthesis/flap failure, and systemic sepsis occurred in fewer than 1.0% of cases. NSQIP-P tracks other postoperative complications including unplanned intubation, pulmonary embolism, venous thrombosis, progressive renal insufficiency, acute renal failure, coma >24 hours, stroke, intraventricular hemorrhage, cardiac arrest requiring CPR, and central line-associated blood infections. There were no incidences of these complications in this study.

**Figure 3:**
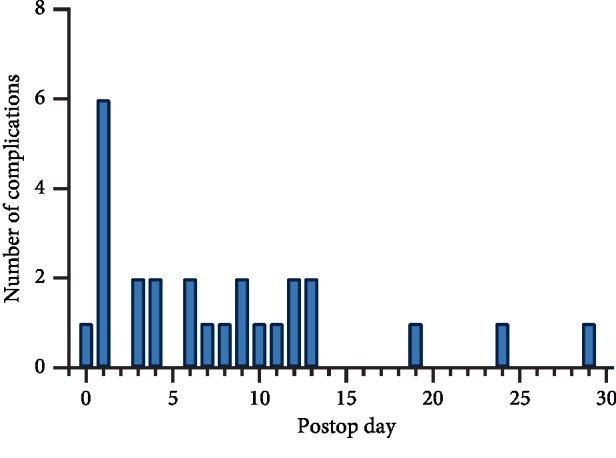
Time to complications.

The overall complication rate, calculated as the number of patients experiencing one or more of the postoperative complications mentioned above, was 8.9%. The majority of complications occurred within the first two weeks following surgery (Figure 3). Complications peaked on postoperative day (POD) 1, with two incidents of deep wound dehiscence, one episode of pneumonia, one seizure, one graft/prosthesis/flap failure, and one nerve injury. After the first postoperative day, the complication rate was steady until POD 14. Only three complications occurred after POD 14, including one incident of superficial wound dehiscence on POD 19, one seizure on POD 24, and one UTI on POD 29.

Fourty-six patients (24.0%) experienced bleeding requiring transfusion during or in the first 72 hours following surgery. Twenty-four patients (12.5%) experienced an unplanned readmission, and seven readmissions were classified as related to the principle procedure (3.6%). Nine patients (4.7%) underwent an unplanned reoperation, with 8 reoperations related to the principle procedure (4.2%). Four patients (2.1%) were still in the hospital thirty days after surgery. The thirty-day mortality rate was low at 1.0% (Table 5). There were no statistically significant differences in postoperative complication rates with respect to tumor origin or location (Table 6). Additionally, there was no association between age and complication or mortality rates.

**Figure 4:**
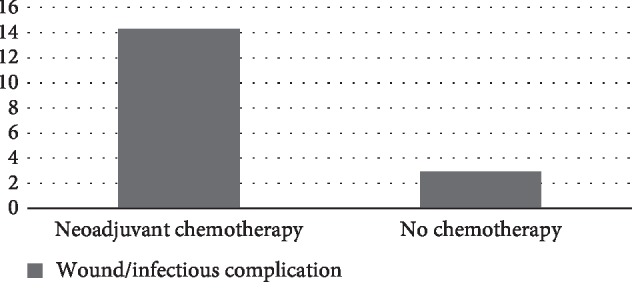
Neoadjuvant chemotherapy increases the incidence of wound/infectious complications.

As a general surgery database, NSQIP-P lacks oncology specific variables. However, between 2012 and 2016, NSQIP-P identified patients who received chemotherapy in the 30 days prior to surgery. These data were available for 96 patients in our cohort. Of those 96 patients, 28 patients (29.2%) received neoadjuvant chemotherapy. NSQIP-P does not provide the precise number of days between neoadjuvant therapy and surgery. Median preoperative WBC counts were significantly lower in patients who received neoadjuvant chemotherapy (4.3 vs 7.2, *p*=0.0004). Additionally, these patients experienced higher rates of postoperative wound and infectious complications (OR 5.5, 95% CI: 1.192–29.73) (Figure 4).

The rate of wound and infectious complications was also related to anesthesia time. Patients who experienced wound or infectious complications had significantly longer anesthesia time than patients who did not experience these complications (465 vs 298 minutes, *p*=0.0003). We also considered the effect of preoperative albumin on wound healing; however, we did not find a significant association between low albumin and poor wound healing (*p*=0.544).

## 4. Discussion

Pediatric bone and soft tissue sarcomas are rare tumors requiring aggressive, multidisciplinary treatment. Large national databases and international collaborations are essential tools for studying epidemiology, outcomes, and trends in order to develop treatment guidelines and guide future research. Oncology registries such as SEER provide excellent data on long-term prognosis and survival trends but fail to capture short-term treatment specific morbidity [13–20]. NSQIP-P is a large, national registry capturing patient demographics, comorbidities, and perioperative complications, making it an ideal tool to study patient characteristics and short-term outcomes after surgical management of sarcomas.

In this study, we identified 192 pediatric patients undergoing surgery for primary bone or soft tissue sarcomas. Soft tissue sarcoma patients were younger than bone sarcoma patients (12 vs 7.5 years, *p*=0.0006). All subgroups were associated with a slight male predominance. These demographics reflect the current epidemiology of childhood sarcomas [1, 2]. However, the relative proportions of bone versus soft tissue sarcomas and axial versus appendicular tumor locations are not representative of national rates [2]. This disparity is due to the fact that NSQIP-P only captures patients treated with surgery. Often Ewing Sarcoma not amenable to surgery are managed with chemotherapy and radiation. Additionally, soft tissue sarcomas, specifically rhabdomyosarcomas, are disproportionately affected because they are more common in the axial skeleton where resection can be limited by proximity of vital organs and neural structures to the tumor making medical management with radiation therapy the preferred treatment [2–4]. Therefore, soft tissue sarcomas, Ewing Sarcoma, and axial tumor locations are under-represented in this study due to treatment without surgery.

Despite the potential for selection bias skewing the axial group towards less complicated resections, axial tumors were associated with higher WBC and platelet counts. A closer examination of WBC and platelet count distributions show that WBC and platelet counts are more strongly skewed to the right in patients with axial tumors. However, the difference appears to be small, and the medians of both groups fall within normal reference ranges. Axial tumors were also more likely to be classified as emergent or urgent (OR 16.98, 95% CI: 2.660–181.9) and had longer median hospital stays. These differences were not seen between the bone and soft tissue sarcoma groups, and the reason for these disparities and their clinical significance is unclear.

On the other hand, patients with appendicular tumors were more likely to have a history of prior childhood malignancy (OR 8.313, 95% CI: 2.146–37.25). We hypothesize that this may reflect patients with tumor predisposition syndromes such as Li-Fraumeni and retinoblastoma. While these patients are susceptible to sarcomas in any location, surgical management is more likely in the appendicular skeleton, while unresectable axial tumors are more likely to be treated with radiotherapy. Because our study only follows surgically treated patients, the prior history of childhood malignancy in the appendicular group is artificially inflated.

We found a higher proportion of African American patients with soft tissue versus bone sarcomas (OR 4.626, 95% CI: 1.350–12.95). A SEER study by Ellis et al. found that African American race was an independent predictor of poor ten-year disease specific survival for head and neck Ewing Sarcoma [37]. Another SEER study by Duchman et al. examined socioeconomic determinants of survival in patients with primary osseous Ewing sarcoma. While socioeconomic status and rural versus urban county were not associated with poor outcomes, they found that African American race was associated with higher rates of metastatic disease at diagnosis and lower disease-specific survival on univariate analysis. However, black race was not an independent risk factor in the multivariate analysis, suggesting that metastatic disease at diagnosis was driving lower disease-specific survival rates [38]. The association between race, tumor biology, and early metastasis warrants additional investigation.

This study demonstrates that pediatric bone and soft tissue sarcoma surgery is associated with significant rates of complications (8.9%), transfusions (24.0%), unplanned readmissions (12.5% overall and 3.6% related), and unplanned reoperations (4.7% overall and 4.2% related). The most frequent complications were wound disruptions and infectious etiologies including SSI, pneumonia, UTI, *C. diff* colitis, and sepsis. Overall, 7.8% of patients in our cohort experienced either wound disruption or an infectious complication. These adverse events are particularly concerning for sarcoma patients who are frequently immunocompromised due to neoadjuvant therapy. Although neoadjuvant chemotherapy data were only available for half of our study patients, we did find a statistically significant difference in WBC count, wound disruption, and infectious complications in patients who received chemotherapy. Subsequent treatment may be delayed in these patients in order to clear an infection or allow an incision to heal.

Postoperative wound and infectious complication rates in sarcoma patients vary widely in the current literature. Li et al. reported a series of 53 osteosarcoma patients where 7.5% of patients experienced wound complications requiring revision surgery and 1.9% developed a prosthetic infection [8]. Lozano-Calderón et al. reported another series of 33 pediatric tibia sarcomas treated with allograft reconstruction. The wound dehiscence rate was 48%, with all but one patient requiring irrigation and debridement [6]. A large cohort of 121 pediatric and adult bone and soft tissue sarcomas from Peel et al. also reported high rates of infectious complications with 16% of patients developing early postoperative bacteremia and 14% of patients developing a tumor endoprosthesis infection [9]. The wide range of complication rates in the literature can be explained by the prolonged follow-up of prior studies. Our thirty-day wound dehiscence and infectious complication rate of 7.8% is significant, but higher rates in studies with longer follow-ups make it clear that infectious complications continue to be a concern well beyond the early postoperative recovery period. Of note, the wound dehiscence rate and surgical site infection rate are higher in the pediatric population compared with our adult sarcoma cohort, 7.8% vs 5.6%, respectively [25]. Potentially, this is related to pressure from the medical oncologists to get patients back on chemotherapy as soon as possible.

In this study, the thirty-day mortality after pediatric bone and soft tissue sarcoma surgery was 1.0%. This is 25-fold higher than the thirty-day mortality rate of 0.04% for all pediatric orthopedic procedures reported to NSQIP-P in 2012 [26]. However, it is lower than the 3.3% mortality rate of adults undergoing spinal tumor resections in the adult NSQIP database between 2011 and 2014 [22]. Additionally, in our previous NSQIP study of adult bone and soft tissue sarcomas of the extremities, we found a thirty-day mortality rate of 0.4% [25]. When our pediatric sarcoma cohort is partitioned by tumor location, axial tumors had a 2.1% thirty-day mortality rate and there were no deaths in the appendicular tumor group. Although this difference did not reach statistical significance, this finding does fit well with the previous studies. Overall, these studies suggest that early mortality is higher with spine tumors than with tumors of the extremities.

### 4.1. Limitations

This study has several limitations. Although large national registries are essential tools for orthopedic oncology research, this study is inherently limited to the data provided in the NSQIP-P database. NSQIP-P is a surgical database, so it cannot be used to characterize outcomes in patients managed nonoperatively. This limitation is especially important when considering data on axial tumors, where surgery may be limited or impossible due to the proximity of vital structures. Some important surgical parameters, such as DVT or antibiotic prophylaxis, are missing from NSQIP-P and are likely heterogenous due to variation in protocols between providers and participating institutions. Additionally, there is a lack of granularity with respect to oncology specific variables such as tumor histology, stage at diagnosis, neoadjuvant or adjuvant treatment, and surgical margin status. NSQIP-P does not track tumor predisposition syndromes or molecular profiles of individual tumors, limiting our ability to stratify cases with unique natural histories and treatment considerations. Furthermore, NSQIP-P only follows patients for the first thirty days following surgery. Complications occurring outside this window are not reflected in the database. Additional research is needed to characterize midterm and patient-centered outcomes such as implant stability, functional status, and return to activities. Finally, this study is limited by a small sample size, diminishing the power of the analysis. Although our scope was broad in an attempt to build a cohort with sufficient statistical power, the case volume in NSQIP-P is currently too small to support a robust analysis of such a rare procedure.

### 4.2. Conclusions

Approximately one in eleven patients will experience a complication in the first thirty days following surgical management of pediatric bone and soft tissue sarcomas. However, early perioperative mortality remains low. Although there are limitations to our analysis, this study represents the first comprehensive review of pediatric bone and soft tissue sarcoma surgery in the NSQIP-P database. As the case volume of NSQIP-P continues to grow, NSQIP-P has the potential to become a powerful tool for pediatric orthopedic oncology research.

## Figures and Tables

**Table 1:** Demographics and comorbidities.

	All patients *N* = 192 *n* (%)
Gender	
Male	110 (57.3%)
Female	82 (42.7%)
Race	
White	147 (76.6%)
Black or African American	13 (6.8%)
Asian or Pacific Islander	7 (3.6%)
Unknown/not reported	25 (13.0%)
Ethnicity	
Hispanic	28 (14.6%)
Non-Hispanic	148 (77.1%)
Unknown/not reported	16 (8.3%)
Preop SIRS	3 (1.6%)
Inotropic support	1 (0.5%)
Nutritional support	8 (4.2%)
Recent steroid use	23 (12.0%)
Cardiac risk factors	
Major cardiac risk factors	3 (1.6%)
Minor cardiac risk factors	5 (2.6%)
History of pulmonary disease	23 (12.0%)
Gastrointestinal disease	7 (3.6%)
CNS disorder	26 (13.5%)
Neuromuscular disorder	10 (5.2%)
Hematologic disorder	26 (13.5%)
Previous childhood malignancy	17 (8.9%)

**Table 2:** Demographics and comorbidities with respect to tumor origin and location.

		Soft tissue *N* = 54 Median (IQR)	Bone *N* = 138 Median (IQR)	*p* value	Axial *N* = 94 Median (IQR)	Appendicular *N* = 98 Median (IQR)	*p* value
Age, years		7.5 (3–12.25)	12 (7–14)	**0.0006**	9 (3.75–12.25)	12 (10–14)	**<0.0001**
		*n* (%)	*n* (%)		*n* (%)	*n* (%)	
Gender							
Male		31 (57.4%)	79 (57.2%)	>0.9999	54 (57.4%)	56 (57.1%)	>0.9999
Female		23 (42.6%)	59 (42.8%)		40 (42.6%)	42 (42.9%)	
Race^a^							
White		37 (68.5%)	110 (79.7%)	**0.0096**	66 (70.2%)	81 (82.7%)	0.158
Black or African American		8 (14.8%)	5 (3.6%)		9 (9.6%)	4 (4.1%)	
Asian or Pacific Islander		2 (3.7%)	5 (3.6%)		4 (4.3%)	3 (3.1%)	
Unknown/not reported		7 (13.0%)	18 (13.0%)		15 (16.0%)	10 (10.2%)	
Ethnicity						
Hispanic		6 (11.1%)	22 (15.9%)	0.762	19 (20.2%)	9 (9.2%)	0.065
Non-Hispanic		43 (79.6%)	105 (76.1%)		66 (70.2%)	82 (83.7%)	
Unknown/not reported		5 (9.3%)	11 (8.0%)		9 (9.6%)	7 (7.1%)	
Preop SIRS		0	3 (2.2%)	0.560	0	3 (3.1%)	0.246
Inotropic support		0	1 (0.7%)	>0.9999	1 (1.1%)	0	0.490
Nutritional support		1 (1.9%)	7 (5.1%)	0.446	4 (4.3%)	4 (4.1%)	>0.9999
Recent steroid use		3 (5.6%)	20 (14.5%)	0.136	14 (14.9%)	9 (9.2%)	0.269
Cardiac risk factors^b^							
Major cardiac risk factors		0	3 (2.2%)	0.446	2 (2.1%)	1 (1.0%)	>0.9999
Minor cardiac risk factors		1 (1.9%)	4 (2.9%)		2 (2.1%)	3 (3.1%)	
History of pulmonary disease		10 (18.5%)	13 (9.4%)	0.089	10 (10.6%)	13 (13.3%)	0.659
Gastrointestinal disease		0	7 (5.1%)	0.194	4 (4.3%)	3 (3.1%)	0.717
CNS disorder		6 (11.1%)	20 (14.5%)	0.643	22 (23.4%)	4 (4.1%)	**<0.0001**
Neuromuscular disorder		3 (5.6%)	7 (5.1%)	>0.9999	8 (8.5%)	2 (2.0%)	0.054
Hematologic disorder		6 (11.1%)	20 (14.5%)	0.643	11 (11.7%)	15 (15.3%)	0.530
Previous childhood malignancy		4 (7.4%)	13 (9.4%)	0.783	2 (2.1%)	15 (15.3%)	**0.0016**

*p* values calculated using Mann–Whitney *U* test for continuous variables and Fisher's exact test or *χ*^2^ test for categorical variables. Significant values are in bold. ^a^Calculated using Fisher's exact test with African American versus other races. ^b^Calculated using Fisher's exact test with major or minor cardiac risk factors compared to no risk factors.

**Table 3:** Preoperative labs and surgery parameters.

	All patients *N* = 192 Median (IQR)
Pre-op labs	
WBC	6.7 (4.4–9.0)
Hematocrit	34 (30.2–38.1)
Platelets	250 (184.0–326.0)
Sodium	139 (137.0–141.0)
BUN	9.5 (7.0–13.0)
Creatinine	0.49 (0.36–0.60)
Albumin	4.2 (3.8–4.4)
Duration of anesthesia (minutes)	303.5 (181.5–435.5)
Length of hospital stay (days)	5 (2–8)

	*n* (%)

ASA class	
ASA 1—no disturbance	6 (3.1%)
ASA 2—mild disturbance	61 (31.8%)
ASA 3—severe disturbance	118 (61.5%)
ASA 4—life threatening	6 (3.1%)
None assigned	1 (0.5%)
Anesthesia	
General	191 (99.5%)
Spinal	1 (0.5%)
Open vs minimally invasive	
Open only	155 (80.7%)
Laparoscopic/MIS only	16 (8.3%)
Open and MIS	6 (3.1%)
Not reported	15 (7.8%)
Inpatient/outpatient	
Inpatient	160 (83.3%)
Outpatient	32 (16.7%)
Emergency surgery	
Elective	177 (92.2%)
Emergent	7 (3.6%)
Urgent	8 (4.2%)

**Table 4:** Preoperative labs and distinct surgery parameters with respect to tumor location.

	Axial *N* = 94 Median (IQR)	Appendicular *N* = 98 Median (IQR)	*p* value
Preoperative labs			
Hematocrit	34.2 (31.0–38.6)	33.7 (29.6–38.5)	0.573
WBC	7 (5.2–10.4)	5.9 (3.8–7.8)	**0.004**
BUN	10 (7.0–13.1)	9 (6.3–12.8)	0.161
Creatinine	0.45 (0.30–0.60)	0.5 (0.40–0.60)	0.170
Sodium	139 (137.0–141.3)	139 (137.0–141.0)	0.592
Platelets	270 (220.3–342.0)	216 (153.5–293.0)	**0.001**
Duration of anesthesia (minutes)	292 (160.8–422.3)	323 (189.3–450.3)	0.367
Length of hospital stay (days)	6 (3–10)	4 (2–7)	**0.051**

	*n* (%)	*n* (%)	

Emergency surgery?			
Elective	80 (85.1%)	97 (99.0%)	**0.0003**
Emergent or urgent	14 (14.9%)	1 (1.0%)	

*p* values calculated using Mann–Whitney *U* test for continuous variables and Fisher's exact test or *χ*^2^ test for categorical variables. Significant values are in bold.

**Table 5:** Postoperative complications.

	All patients *N* = 192 *n* (%)
Surgical site infection	
Superficial	3 (1.6%)
Deep	2 (1.0%)
Wound dehiscence	
Superficial	5 (2.6%)
Deep	2 (1.0%)
Pneumonia	3 (1.6%)
UTI	3 (1.6%)
Seizure	3 (1.6%)
Nerve injury	1 (0.5%)
Graft/prosthesis/flap failure	1 (0.5%)
*C. Diff*	2 (1.0%)
Systemic sepsis	1 (0.5%)
One or more complication^a^	17 (8.9%)
Bleeding requiring transfusion^b^	46 (24.0%)
Unplanned readmission	24 (12.5%)
Related	7 (3.6%)
Unrelated	17 (8.9%)
Unplanned reoperation	9 (4.7%)
Related	8 (4.2%)
Unrelated	1 (0.5%)
Still in hospital >30 days	4 (2.1%)
Death	2 (1.0%)

^a^One or more complication refers to the number of patients with one or more of the complications listed above. ^b^Bleeding requiring transfusion defined as intraoperative transfusion or transfusion given within the first 72 hours after surgery.

**Table 6:** Postoperative complications with respect to tumor origin and location.

	Soft tissue *N* = 54 *n* (%)	Bone *N* = 138 *n* (%)	*p* value	Axial *N* = 94 *n* (%)	Appendicular *N* = 98 *n* (%)	*p* value
Surgical site infection						
Superficial	1 (1.9%)	2 (1.4%)	>0.9999	1 (1.1%)	2 (2.0%)	0.369
Deep	0	2 (1.4%)		0	2 (2.0%)	
Wound dehiscence						
Superficial	1 (1.9%)	4 (2.9%)	0.675	2 (2.1%)	3 (3.1%)	0.445
Deep	0	2 (1.4%)		0	2 (2.0%)	
Pneumonia	1 (1.9%)	2 (1.4%)	>0.9999	3 (3.2%)	0	0.115
UTI	0	3 (2.2%)	0.560	1 (1.1%)	2 (2.0%)	>0.9999
Seizure	1 (1.9%)	2 (1.4%)	>0.9999	2 (2.1%)	1 (1.0%)	0.615
Nerve injury	0	1 (0.7%)	>0.9999	0	1 (1.0%)	>0.9999
Graft/prosthesis/flap failure	1 (1.9%)	0	0.281	0	1 (1.0%)	>0.9999
*C. Diff*	1 (1.9%)	1 (0.7%)	0.485	2 (2.1%)	0	0.238
Systemic sepsis	0	1 (0.7%)	>0.9999	1 (1.1%)	0	0.490
One or more complication^a^	3 (5.6%)	14 (10.1%)	0.405	8 (8.5%)	9 (9.2%)	>0.9999
Bleeding requiring transfusion^b^	13 (24.1%)	33 (23.9%)	>0.9999	17 (18.1%)	29 (29.6%)	0.066
Unplanned readmission	8 (14.8%)	16 (11.6%)	0.628	12 (12.8%)	12 (12.2%)	>0.9999
Related	2 (3.7%)	5 (3.6%)	>0.9999	4 (4.3%)	3 (3.1%)	0.717
Unrelated	6 (11.1%)	11 (8.0%)	0.573	8 (8.5%)	9 (9.2%)	>0.9999
Unplanned reoperation	3 (5.6%)	6 (4.3%)	0.713	5 (5.3%)	4 (4.1%)	0.744
Related	3 (5.6%)	5 (3.6%)	0.689	4 (4.3%)	4 (4.1%)	>0.9999
Unrelated	0	1 (0.7%)	>0.9999	1 (1.1%)	0	0.490
Still in hospital >30 days	1 (1.9%)	3 (2.2%)	>0.9999	2 (2.1%)	2 (2.0%)	>0.9999
Death	1 (1.9%)	1 (0.7%)	0.485	2 (2.1%)	0	0.238

*p* values calculated using Mann–Whitney *U* test for continuous variables and Fisher's exact test or *χ*^2^ test for categorical variables. Significant values are in bold. ^a^One or more complication refers to the number of patients with one or more of the complications listed above. ^b^Bleeding requiring transfusion defined as intraoperative transfusion or transfusion given within the first 72 hours after surgery.

## Data Availability

The patient data used to support the findings of this study are restricted to employees of ACS NSQIP-participating hospitals. Data are available from the ACS NSQIP website (https://www.facs.org/quality-programs/acs‐nsqip/participant-use) for researchers who meet the criteria for access to the program database.

## Supplementary Materials

Supplementary Materials Appendix A: ICD-9 and ICD-10 codes used for patient identification. Appendix B: CPT codes used for patient identification. Appendix C: CPT codes used as exclusion criteria.
