# Supplementary material for: Thirty-Day Outcomes following Pediatric Bone and Soft Tissue Sarcoma Surgery: A NSQIP Pediatrics Analysis
Source: Sarcoma. 2020 Feb 14;2020:1283080. doi: 10.1155/2020/1283080 (PMC7042554; doi:10.1155/2020/1283080)
Supplement: Supplementary Materials — Appendix A: ICD-9 and ICD-10 codes used for patient identification. Appendix B: CPT codes used for patient identification. Appendix C: CPT codes used as exclusion criteria. [file 1283080.f1.docx]

**Appendix A:** ICD-9 and ICD-10 codes used for patient identification

| **ICD-9** | **ICD-10** | **Diagnosis** |
| --- | --- | --- |
| 170.4 | C40.00 - .02 | Malignant neoplasm of scapula and long bones of upper limb |
| 170.5 | C40.10 - .12 | Malignant neoplasm of short bones of upper limb |
| 170.7 | C40.20 - .22 | Malignant neoplasm of long bones of lower limb |
| 170.8 | C40.30 - .32 | Malignant neoplasm of short bones of lower limb |
|  | C40.80 - .82 | Malignant neoplasm of overlapping sites of bone and articular cartilage of limb |
|  | C40.90 - .92 | Malignant neoplasm of unspecified bones and articular cartilage of limb |
| 170.0 | C41.0 | Malignant neoplasm of bones of skull and face |
| 170.1 | C41.1 | Malignant neoplasm of mandible |
| 170.2 | C41.2 | Malignant neoplasm of vertebral column |
| 170.3 | C41.3 | Malignant neoplasm of ribs, sternum, and clavicle |
| 170.6 | C41.4 | Malignant neoplasm of pelvic bones, sacrum, and coccyx |
| 170.9 | C41.9 | Malignant neoplasm of bone and articular cartilage, unspecified |
|  | C47.0 | Malignant neoplasm of peripheral nerves of head, face, and neck |
|  | C47.10 - .12 | Malignant neoplasm of peripheral nerves of upper limb, including shoulder |
|  | C47.20 - .22 | Malignant neoplasm of peripheral nerves of lower limb, including hip |
|  | C47.3 | Malignant neoplasm of peripheral nerves of thorax |
|  | C47.4 | Malignant neoplasm of peripheral nerves of abdomen |
|  | C47.5 | Malignant neoplasm of peripheral nerves of pelvis |
|  | C47.6 | Malignant neoplasm of peripheral nerves of trunk, unspecified |
|  | C47.8 | Malignant neoplasm of overlapping sites of peripheral and autonomic nervous system |
|  | C47.9 | Malignant neoplasm of peripheral nerves and autonomic nervous system, unspecified |
| 171.0 | C49.0 | Malignant neoplasm of connective and soft tissue of the head, face, and neck |
| 171.2 | C49.10 - .12 | Malignant neoplasm of connective and soft tissue of the upper limb, including shoulder |
| 171.3 | C49.20 - .22 | Malignant neoplasm of connective and soft tissue of the lower limb, including hip |
| 171.4 | C49.3 | Malignant neoplasm of connective and soft tissue of the thorax |
| 171.5 | C49.4 | Malignant neoplasm of connective and soft tissue of the abdomen |
| 171.6 | C49.5 | Malignant neoplasm of connective and soft tissue of the pelvis |
| 171.7 | C49.6 | Malignant neoplasm of connective and soft tissue of the trunk, unspecified |
| 171.8 | C49.8 | Malignant neoplasm of overlapping sites of connective and soft tissue |
| 171.9 | C49.9 | Malignant neoplasm of connective and soft tissue, unspecified |

**Appendix B:** CPT codes used for patient identification

| Amputation | | | | | | | |
| --- | --- | --- | --- | --- | --- | --- | --- |
| 23900 | 23920 | 24900 | 24920 | 24931 | 25900 | 25905 | 25920 |
| 25927 | 26910 | 26951 | 26952 | 27290 | 27295 | 27590 | 27591 |
| 27592 | 27594 | 27596 | 27598 | 27880 | 27881 | 27882 | 27888 |
| 27889 | 28800 | 28805 | 28810 | 28820 | 28825 |  |  |
| Arthroplasty | | | | | | | |
| 21240 | 21242 | 21243 | 23470 | 23472 | 24360 | 24361 | 24362 |
| 24363 | 24365 | 24366 | 25332 | 25441 | 25442 | 25443 | 25444 |
| 25445 | 25446 | 25447 | 26530 | 26531 | 26535 | 26536 | 27120 |
| 27122 | 27125 | 27130 | 27418 | 27437 | 27438 | 27440 | 27441 |
| 27442 | 27443 | 27445 | 27446 | 27447 | 27700 | 27702 |  |
| Arthrodesis | | | | | | | |
| 22532 | 22533 | 22534 | 22548 | 22551 | 22552 | 22554 | 22556 |
| 22558 | 22585 | 22586 | 22590 | 22595 | 22600 | 22610 | 22612 |
| 22614 | 22630 | 22632 | 22633 | 22634 | 22800 | 22802 | 22804 |
| 22808 | 22810 | 22812 | 22818 | 22819 | 23800 | 23802 | 24800 |
| 24802 | 25800 | 25805 | 25810 | 25820 | 25825 | 25830 | 26820 |
| 26841 | 26842 | 26843 | 26844 | 26850 | 26852 | 26860 | 26861 |
| 26862 | 26863 | 27280 | 27282 | 27284 | 27286 | 27580 | 27870 |
| 27871 | 28705 | 28715 | 28725 | 28730 | 28735 | 28737 | 28740 |
| 28750 | 28755 | 28760 |  |  |  |  |  |
| Open Reduction Internal Fixation | | | | | | | |
| 21325 | 21330 | 21335 | 21336 | 21338 | 21339 | 21343 | 21344 |
| 21346 | 21347 | 21348 | 21356 | 21360 | 21365 | 21366 | 21385 |
| 21386 | 21387 | 21390 | 21395 | 21406 | 21407 | 21408 | 21422 |
| 21423 | 21445 | 21454 | 21461 | 21462 | 21465 | 21470 | 21811 |
| 21812 | 21813 | 21825 | 22325 | 22326 | 22327 | 22328 | 23515 |
| 23585 | 23615 | 23616 | 23630 | 24545 | 24546 | 24575 | 24579 |
| 24586 | 24587 | 24635 | 24665 | 24666 | 24685 | 25515 | 25525 |
| 25526 | 25545 | 25574 | 25575 | 25607 | 25608 | 25609 | 25628 |
| 25645 | 25652 | 25685 | 26615 | 26665 | 26735 | 26746 | 26765 |
| 26785 | 27202 | 27215 | 27217 | 27218 | 27226 | 27227 | 27228 |
| 27236 | 27248 | 27269 | 27511 | 27513 | 27514 | 27519 | 27524 |
| 27535 | 27536 | 27540 | 27766 | 27769 | 27784 | 27792 | 27814 |
| 27822 | 27823 | 27826 | 27827 | 27828 | 28415 | 28420 | 28445 |
| 28450 | 28455 | 28465 | 28485 | 28505 | 28525 | 28531 | 24515 |
| 27244 | 27507 | 27758 | 24516 | 27245 | 27506 | 27759 |  |
| External Fixation | | | | | | | |
| 20650 | 20662 | 20663 | 20690 | 20692 | 20696 | 20697 | 21100 |
| Prophylactic Stabilization | | | | | | | |
| 23490 | 23491 | 24498 | 25490 | 25491 | 25492 | 27187 | 27495 |
| 27745 |  |  |  |  |  |  |  |
| Excision, Tumor, Subcutaneous or Subfascial | | | | | | | |
| 21011 | 21012 | 21552 | 21555 | 21930 | 21931 | 22902 | 22903 |
| 23071 | 23075 | 24071 | 24075 | 25071 | 25075 | 26111 | 26115 |
| 27043 | 27047 | 27327 | 27337 | 27618 | 27632 | 28039 | 28043 |
| 21013 | 21014 | 21554 | 21556 | 21932 | 21933 | 22900 | 22901 |
| 23073 | 23076 | 24073 | 24076 | 25073 | 25076 | 26113 | 26116 |
| 27045 | 27048 | 27328 | 27339 | 27619 | 27634 | 28041 | 28045 |
| Radical Resection, Tumor, Soft Tissue (e.g. sarcoma) or Bone | | | | | | | |
| 21015 | 21016 | 21557 | 21558 | 21935 | 21936 | 22904 | 22905 |
| 23077 | 23078 | 24077 | 24079 | 25077 | 25078 | 26117 | 26118 |
| 27049 | 27059 | 27329 | 27364 | 27615 | 27616 | 28046 | 28047 |
| 19260 | 19271 | 21034 | 21044 | 21045 | 23200 | 23210 | 23220 |
| 24150 | 24152 | 25170 | 26250 | 26260 | 26262 | 27075 | 27076 |
| 27077 | 27078 | 27365 | 27640 | 27641 | 27645 | 27646 | 27647 |
| 28171 | 28173 | 28175 | 61500 |  |  |  |  |
| Resection, Bone | | | | | | | |
| 21050 | 21070 | 21600 | 21615 | 21620 | 21630 | 21632 | 22100 |
| 22101 | 22102 | 22103 | 22110 | 22112 | 22114 | 22116 | 23120 |
| 23125 | 23190 | 23195 | 24130 | 24155 | 25210 | 25215 | 25240 |
| 26185 | 27080 | 27350 | 28111 | 28112 | 28113 | 28114 | 28118 |
| 28126 | 28130 | 28140 | 28150 | 28153 | 28160 | 31254 | 31287 |
| 31288 | 32900 | 69501 | 63285 | 63286 | 63287 | 63290 |  |
| Osteoplasty | | | | | | | |
| 23130 | 24420 | 25390 | 25391 | 25392 | 25393 | 25394 | 26568 |
| 27465 | 27466 | 27468 | 27715 |  |  |  |  |
| Osteotomy | | | | | | | |
| 21198 | 21199 | 21206 | 22206 | 22207 | 22208 | 22210 | 22212 |
| 22214 | 22216 | 22220 | 22222 | 22224 | 22226 | 23480 | 23485 |
| 24400 | 24410 | 25350 | 25355 | 25360 | 25365 | 25370 | 25375 |
| 26565 | 26567 | 27140 | 27146 | 27147 | 27151 | 27156 | 27161 |
| 27165 | 27448 | 27450 | 27454 | 27455 | 27457 | 27705 | 27707 |
| 27709 | 27712 | 28304 |  |  |  |  |  |
| Arthrotomy | | | | | | | |
| 23100 | 23101 | 23105 | 23106 | 23107 | 24100 | 24101 | 24102 |
| 25100 | 25101 | 25105 | 25107 | 26100 | 26105 | 26110 | 27050 |
| 27052 | 27054 | 27330 | 27331 | 27332 | 27333 | 27334 | 27335 |
| 27620 | 27625 | 27626 | 28050 | 28052 | 28054 |  |  |
| Epiphyseal Procedure | | | | | | | |
| 20150 | 25450 | 25455 | 27185 | 27475 | 27477 | 27479 | 27485 |
| 27730 | 27732 | 27734 | 27740 | 27742 |  |  |  |
| Other Miscellaneous Excision | | | | | | | |
| 21060 | 25109 | 25110 | 26160 | 26170 | 26180 | 27347 | 27630 |
| 28090 | 28092 | 31237 |  |  |  |  |  |
| Tumor Ablation | | | | | | | |
| 20982 | 20983 |  |  |  |  |  |  |
| Biopsy, Bone or Soft Tissue | | | | | | | |
| 20220 | 20225 | 20240 | 20245 | 20200 | 20205 | 20206 | 21550 |
| 21920 | 21925 | 23065 | 23066 | 24065 | 24066 | 25065 | 25066 |
| 27040 | 27041 | 27323 | 27324 | 27613 | 27614 |  |  |

**Appendix C:** CPT codes used as exclusion criteria

| Excision Benign Bone Tumor | | | | | | | |
| --- | --- | --- | --- | --- | --- | --- | --- |
| 21029 | 21030 | 21031 | 21032 | 21040 | 21046 | 21047 | 21048 |
| 21049 | 23140 | 23145 | 23146 | 23150 | 23155 | 23156 | 24110 |
| 24115 | 24116 | 24120 | 24125 | 24126 | 25120 | 25125 | 25126 |
| 25130 | 25135 | 25136 | 26200 | 26205 | 26210 | 26215 | 27065 |
| 27066 | 27067 | 27355 | 27356 | 27357 | 27358 | 27635 | 27637 |
| 27638 | 28100 | 28102 | 28103 | 28104 | 28106 | 28107 | 28108 |
| Excision Benign Soft Tissue Tumor | | | | | | | |
| 11400 | 11402 | 11403 | 11404 | 11406 | 11420 | 11424 | 11426 |
| 11440 | 11441 | 11443 | 11444 |  |  |  |  |
